# Human-water interactions associated to cercarial emergence pattern and their influences on urinary schistosomiasis transmission in two endemic areas in Mali

**DOI:** 10.1186/s40249-024-01229-w

**Published:** 2024-08-29

**Authors:** Bakary Sidibé, Privat Agniwo, Assitan Diakité, Boris Agossou Eyaton-olodji Sègnito Savassi, Safiatou Niaré Doumbo, Ahristode Akplogan, Hassim Guindo, Moudachirou Ibikounlé, Laurent Dembélé, Abdoulaye Djimde, Jérôme Boissier, Abdoulaye Dabo

**Affiliations:** 1 Department of Epidemiology of Infectious Diseases, Faculty of Pharmacy, University of Sciences, Techniques and Technologies of Bamako, IRL 3189 Bamako, Mali; 2 Centre de Recherche Pour La Lutte Contre Les Maladies Infectieuses Tropicales (CReMIT/TIDRC), Université d’Abomey-Calavi, Abomey-Calavi, Bénin; 3 IHPE, Univ. Montpellier, CNRS, Ifremer, Univ. Perpignan Via Domitia, Perpignan, France

**Keywords:** Schistosomiasis, Chronobiology, Cercarial emission, Snail, Water contact, Mali

## Abstract

**Background:**

Mali is known to be a schistosomiasis-endemic country with a limited supply of clean water. This has forced many communities to rely on open freshwater bodies for many human-water contact (HWC) activities. However, the relationship between contact with these water systems and the level of schistosome infection is currently receiving limited attention. This study assessed human-water interactions including cercarial emergence pattern and their influences on urinary schistosomiasis transmission in two communities in the Kayes district of Mali.

**Methods:**

We carried out a parasitological study first in children in September 2021, then a cross-sectional study of quantitative observations of human-water contact activities in the population, and finally a study of snail infectivity at contact points in September 2022. The study took place in two communities, Fangouné Bamanan and Diakalèl in the Kayes region of western Mali. The chronobiological study focused on cercarial release from naturally infected snails. Released cercariae were molecularly genotyped by targeting the cox1 region, and the ITS and 18S ribosmal DNA gene (18S rDNA) regions of the DNA. Links between sociodemographic parameters, human water-contact points and hematuria were established using multivariate statistical analysis or the logistic regression model.

**Results:**

The main factor predisposing the 97 participants to water contact was domestic activity (62.9%). Of the 378 snails collected at 14 sampling sites, 27 (7.1%) excreted schistosome cercariae, with 15.0% (19/126) at Fangouné Bamanan and 3.3% (8/252) at Diakalel. The release of *Schistosoma* cercariae shows three different patterns in Fangouné Bamanan: (i) an early release peak (6:00–8:00 AM), (ii) a mid-day release peak (10:00 AM–12:00 PM) and (iii) a double peak: (6:00–8:00 AM) and (6:00–8:00 PM) cercariae release; and two release patterns in Diakalel: early release (6:00–8:00 AM) and (ii) mid-day release (12:00–2:00 PM). All cercariae released during early diurnal (6:00–8:00 AM) or nocturnal emission patterns (6:00–8:00 PM) were hybrids parasite having an cox1 *S. bovis* or *S. curassoni* associated with an ITS and 18S rDNA of *S. haematobium* while the cercariae released during diurnal, or mid-day patterns (8:00 AM–6:00 PM) were pure *S. haematobium*.

**Conclusions:**

Our study showed that domestic activity is the main source of exposure in the Kayes region. Two and three cercariae emission patterns were observed at Diakalel and Fangouné Bamanan respectively. These results suggest that the parasite adapts to the human-water contact period in order to increase its infectivity.

**Graphical Abstract:**

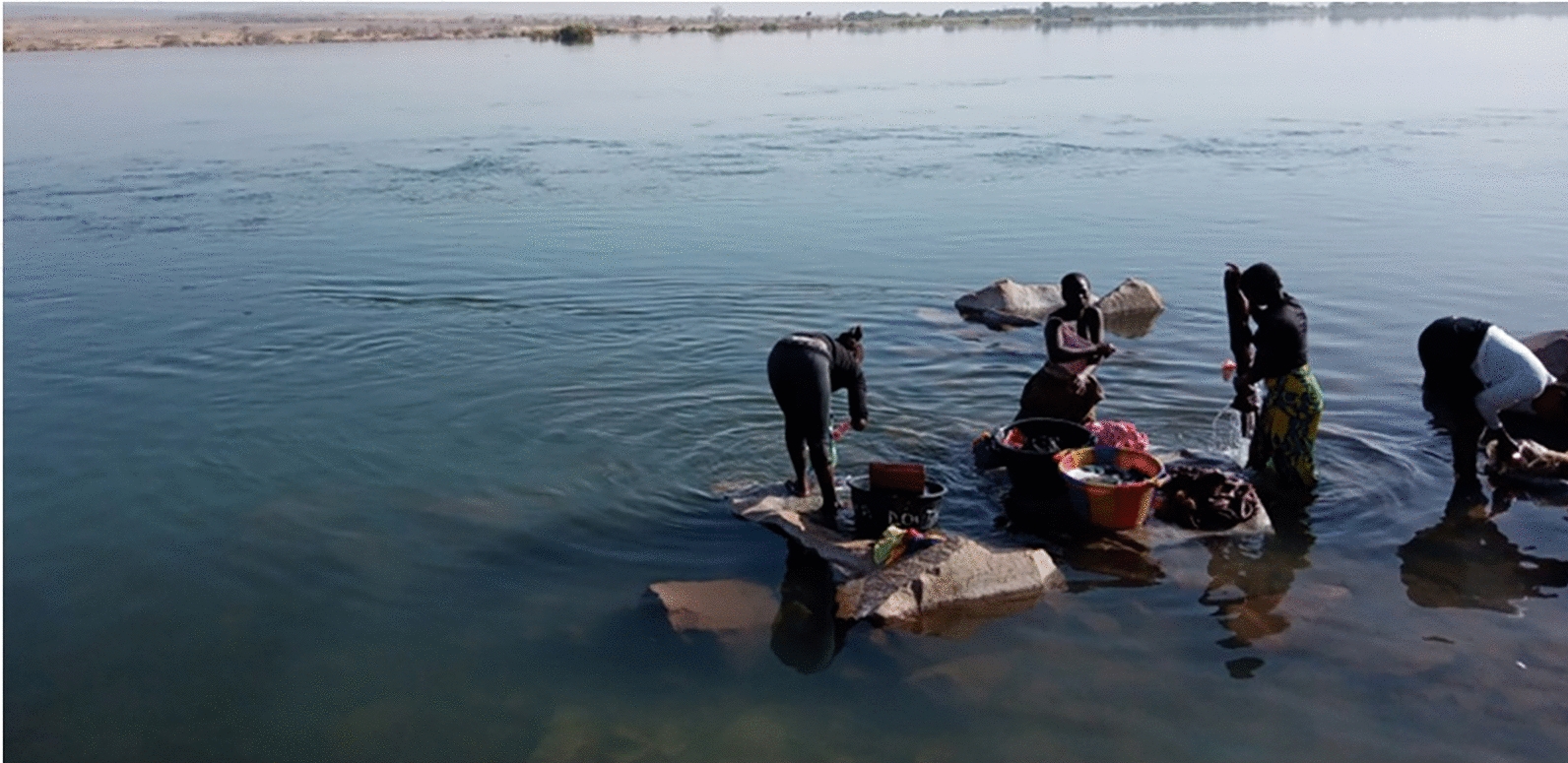

## Background

Schistosomiasis is a widely-recognized parasitic disease of deprived populations in Africa, Asia, and Central and South America, with significant health impact on both human and animal population [1, 2]. This disease affects almost 240 million people worldwide, most of which are found in sub-Saharan Africa [3]. Furthermore, West-African countries, such as Ghana, Mali, Burkina Faso, Côte d’Ivoire, Niger, Senegal and Nigeria are considered to be highly endemic with schistosomiasis [4–10]. The major schistosome species include *Schistosoma haematobium (Sh)* and *S. mansoni (Sm)* [11]. *Sh* accounted for over 85% of cases of urogenital schistosomiasis, while *Sm*, the causal agent of intestinal schistosomiasis, was less prevalent [12, 13].

In Mali, two *Schistosoma* species (*Sh* and *Sm*) have been reported since the first cases were recorded in the 1940s [14]**.** Currently, both forms of the disease occur with geographical variations both in prevalence and intensity [15]. Except for the irrigated rice-growing areas in the “*Office du Niger”* where the two species are co-endemic, *Sh* appears to be the most common species, especially in the Dogon plateau and the Senegal River Basin [16–18]. Schistosomes (*S. bovis, S. curassoni or S. mattheei and rhodaini*) also infect domestic and wild animals like livestock, rodents, etc. The cohabitation of humans with animals in the same environment can lead to hybridization of the schistosome species they host, a phenomenon quite common in many schistosomiasis endemic areas of sub-Saharan Africa [19–22]. The primary snail species implicated in the transmission of human schistosomiasis include *Bulinus* species, *B. truncatus* and *B. globosus* for *Sh*, as well as *Biomphalaria pfeifferi* for *Sm* [23–25]**.**

Transmission of schistosomiasis is significantly influenced by people's behaviors in terms of contacts (swimming, fishing, bathing, washing, and laundry) with water in schistosomiasis endemic areas. In areas where schistosomiasis is endemic, humans have always developed interactions with surface water systems on which they depend, resulting contamination by excreta of water sources and exposure of humans to infectious diseases such as schistosomiasis. Equally important are the presence and distribution of infected intermediate snail hosts within watercourses, which play a crucial role in the disease's transmission dynamics [26]. The schistosome parasite has a complex life cycle that involves two hosts: a freshwater snail, which acts as the intermediate host in which the parasite undergoes larval development, and the definitive hosts (humans or animals) in which the parasite matures into an adult [27, 28]. In order to ensure the survival of the species and the successful transmission of parasites, many trematode species, such as schistosomes, have synchronized their daily emergence rhythms with vertebrate host visits/activity to the biotope [29–32]. The satisfaction of a vital need of animals (watering early between 6:00 to 10:00 AM in the morning before going to pasture or late between 6:00 and 8:00 PM after their return) promotes *S. bovis (Sb)* infestation. In contrast, people's attraction to water during the hot hours between 10:00 AM to 6:00 PM (swimming and recreational activities) induces *Sh* infestation by the opening of the host/parasite encounter filter. So, as snails are intermediate hosts that release the cercarial larvae of schistosomes that infest humans, their examination provides important information on active transmission foci. Because of the possibility of hybridization between human and animal schistosomes, we thought it useful to identify the genetic profiles of cercariae that are released by snails hosts naturally infected at different times of the day. Moreover, as highlighted previously, there has been wide advocacy to integrate water-sanitation and hygiene (WASH), health education, environmental actions and snail control into the mass drug administration (MDA) control strategy [33]. For almost two decades, Mali’s Schistosomiasis National Control Program has consistently adopted the preventive chemotherapy strategies as recommended by the World Health Organization (WHO) [34, 35]. Regrettably, the MDA alone does not offer an effective protection against initial infection or subsequent re-infection in environments contaminated with the disease. The continued transmission of schistosomiasis in regions with a high disease burden, such as Office du Niger, Plateau Dogon, and the Senegal River Basin [7, 36, 37], underscores the necessity for supplementary control strategies that delve into the patterns of human-water contact and the factors that sustain the schistosome lifecycle and its transmission. In redefining WHO's priorities to sustain results, snail control has recently been re-prioritized as a schistosomiasis control strategy to complement MDA [3]. However, a major knowledge gap remains, especially regarding how snail biology and ecology affect schistosomiasis transmission and control outcomes. Despite the interventions, including the MDA, schistosomiasis remains a serious threat to populations, especially in the Office du Niger and Senegal River basin which are recognized as development hubs in the country. Even if the hybrid strains of schistosomes recently described in Mali [19, 38–40] could be involved, schistosomiasis infection, human water contact and snails’ biology are thus essentially linked, and more knowledge about their relationship will help us to develop appropriate control measures. So far, few studies have related water contact patterns to infection levels in Mali. Furthermore. The aim of this study was to explore and examine the influence of human-water interactions, including snail biology, on urinary schistosomiasis transmission in the Senegal River basin in Mali.

## Methods

### Study sites

**Fig. 1:**
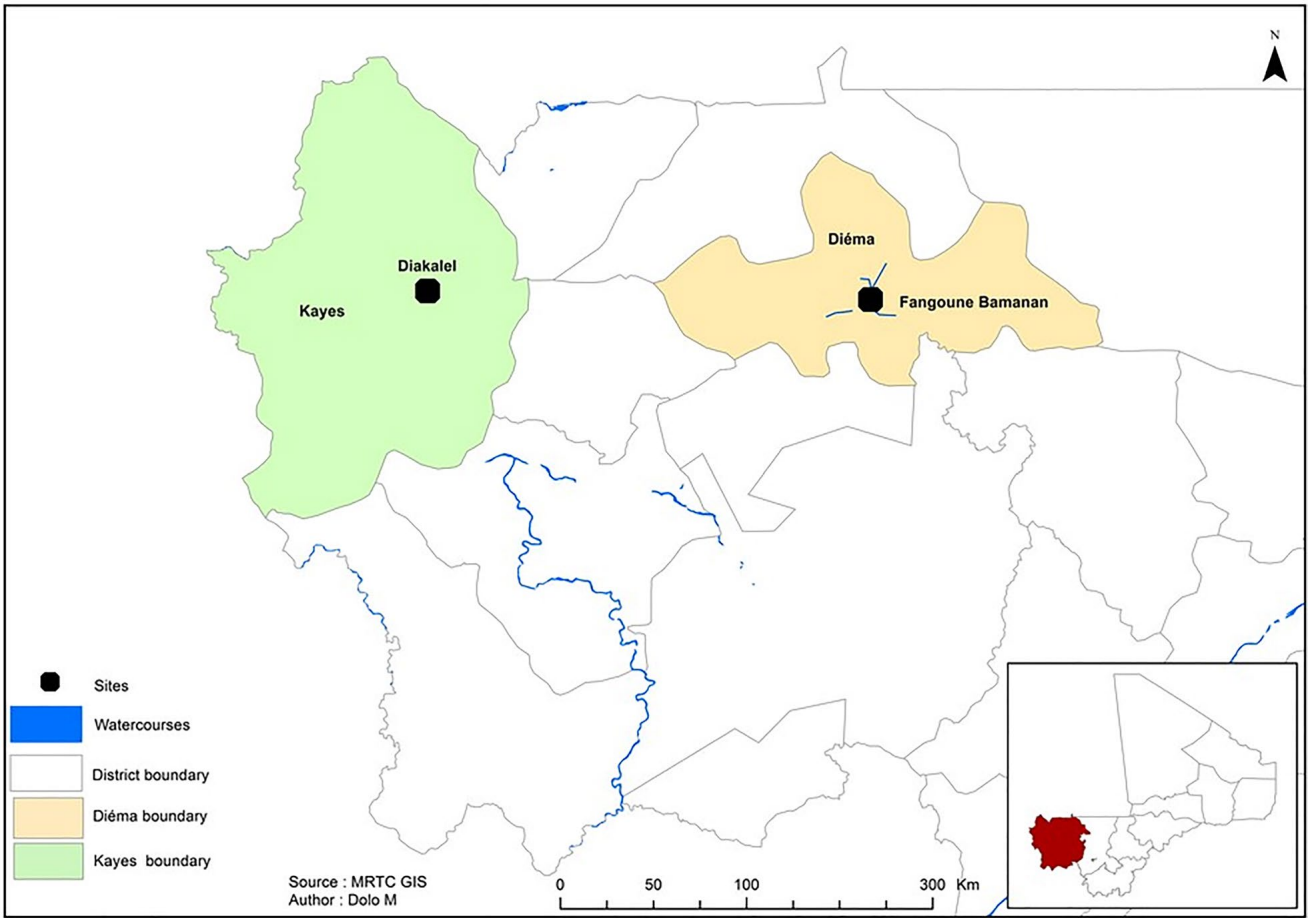
Localization of the two study sites (Diakalel and Fangouné Bamanan) on the map of the Kayes region (Mali, West Africa) in September 2021 and September 2022

We carried out this study in two communities in the Kayes region of western Mali (geographical coordinates between 11°26′40″ W and 14°26′48″ N) known for their endemicity for *Sh *[19]. The two communities surveyed, Fangouné Bamanan (Diéma district) and Diakalel (Kaye district), are 300 km apart (Fig. 1). They were chosen based on their proximity to water sources (ponds in the Diéma district, the Senegal River and its tributaries in the vicinity of the city of Kayes). The Kayes region is characterized by a northern Sudanese climate in the south and a Sahelian climate in the north with two main seasons: the rainy season (June to October) marked by average annual rainfall of up to 1000 mm in the south and 600 to 800 mm in the north, and the dry season which extends from November to April–May [41]. The dry season is divided into hot dry season (March to May) and cold dry season (June to October). The water points created fed by rainwater (ponds and the river tributaries are excellent snail breeding sites). Agriculture and livestock are the two main economic activities of the population [41]. The Sudano-Sahelian climate of the region is indeed favorable to the cultivation and especially to extensive livestock farming where numerous herds of cattle, sheep and goats cohabit. The practice of these two activities around the same water points creates favorable conditions for the mixing of genes between animal and human schistosomes.

### Type of study and parasitological examination of urine

We conducted a cross-sectional and observational study including a parasitological survey in schoolchildren in September 2021. We calculated the minimum sample size on the basis of the previous prevalence (36%) of the disease obtained in each school using the Schwartz formula, taking into account a 10% refusal rate and sampling errors [42]. We selected students in the schools on the basis of simple random sampling from the class list. The names contained in an envelope were drawn at random until the required size was reached. Urine samples were collected from 393 children aged 6–14 years old using sterile containers between 9:00 AM and 2:00 PM. Each child was assigned an identification number based on the first two letters of the village name. Once the urine was homogenized in the jar, a 10 ml was taken with a syringe and filtered through a numbered Whatman filter paper (diameter 25 mm) previously placed in a filter holder. The filtrate was then stained with 3% ninhydrin, dried and rewetted with tap water and then viewed under a compound microscope with either × 4 or × 10 objective for *Sh* eggs. The WHO standard was employed in determining the prevalence and intensity (Low: 1–49 egg/10 ml of urine; High: ≥ 50 egg/10 ml of urine) of schistosomiasis respectively. Ten percent (10%) of the filtrate were re-examined by a senior parasitologist for quality control. All schoolchildren infected by schistosomiasis were verbally questioned on the water sites frequented in order to search for the snail vectors and evaluate the parameters (cercariae release time from infected snails, human-water contact time, etc.) favouring infection of the population.

### Human water contact interactions and exposure risk

A human water contact survey coupled to malacological prospections conducted during the cross-sectional study in September 2022. The information gathered from infected schoolchildren was used to select specific areas for malacological surveys and analysis of human-water contact times. During the observation of contacts with water, structured questionnaires were administered in the local “Bambanakan” language to participants by a socio-anthropologist. Information on their water contact habits, access to drinking water, sanitation and hygiene facilities, self-reported experiences of schistosomiasis, perceptions of exposure and risk factors, and the presence of blood in urine were recorded. We opted for an exhaustive study, including all people who encountered water during our study period and who gave their consent. The study included all the villagers regardless of ages and sexes living in the study areas.

**Fig. 2:**
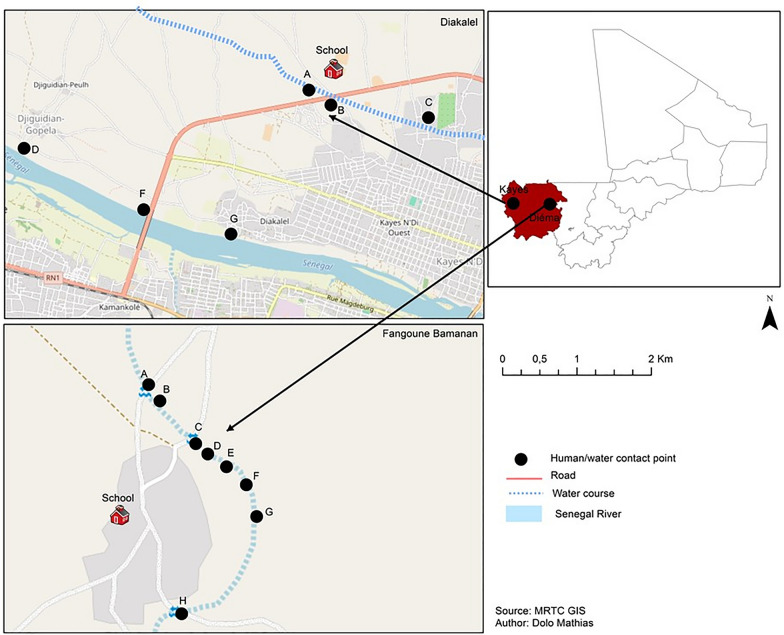
Map of sampling human-water contact points (HWCP) in the two study sites (Diakalel and Fangouné Bamanan), September 2022. Alphabetical letters (A, B, C, D, E, F, G et H) denote HWCP at each site

During the study of human-water contact, we carried out on sites observations of activities promoting people's contact with water at the main contact points. The duration of contact with water was taken by the interviewer using a stopwatch. Concerning the study of the human-water contact, we carried out on-site observations of activities promoting people's contact with water at the main contact points. The observations were made during one week in September 2022 (rainy season). Six human water-contact points (HWCP, A to F) in Diakalel and eight (A to H) in Fangouné Bamanan were surveyed over the three months (Fig. 2).

The main information collected were age, sex, body part and duration of water contact activities. The activities requiring contact with water that we identified in each community have been classified into three main categories: domestic (washing kitchen utensils, laundry, fetching water), occupational (fishing, crossing water, watering animals) and recreational (bathing, swimming, playing). Water contact activity in snail-infested water (intermediate host) was defined as exposure.

### Water contact duration, water-contact frequency, and water contact activities

Having any water contact was defined as a binary measure (i.e., whether the respondent has had contact with one of the freshwater sites we examined during the period of our study). Contact parameters were recorded according to method described previously [43]. Indeed, duration of water-contact was defined as a measure of how long the individual was in contact with the water during per exposure event in the study; water-contact frequency was defined as a measure indicating the number of times per day, per week the respondent was in contact with any of the freshwater we have studied. And water-contact activities were defined as measures of whether the respondent engaged in a given water-contact activity, such as recreational, domestic or professional.

### Geographical distribution of snails, snail sampling and cercarial management

To determine the geographical distribution of snail’s intermediate hosts, all sampled habitats were mapped using hand-held differential geographic global positioning system (GPS) units (Trimble Navigation Ltd, California, USA) with an estimated accuracy of ± 1 m. Data were downloaded with differential correction into a GPS database (GPS pathfinder office 2.8 Trimble Navigation Ltd, California, USA) and analyses performed using ArcView version 9.2 software (Environmental Systems Research Institute, Inc., Redlands, CA).

We conducted collection of snail intermediate hosts in two communities: Fangouné Bamanan and Diakalel, at the same points of human water contact activities (Fig. 2). Snail sampling was conducted by two field sample collectors throughout the study using standard snail sieves or occasionally, by hand picking using long pliers on rocks, rags, old mats, cans, etc. Sampling time was about 15 min per HWCP and was performed between 9:00 AM and 12:00 PM during rainy and cold dry season and between 8:00 AM and 11:00 AM in hot dry season. Sampling area per HWCP varied approximately 3 m^2^ to 5 m^2^ according to the surface to be examined. At each collection time, snails from each site were appropriately labelled and transported in separate perforated plastic in Kayes or in Diema, where they were processed. Snails were identified to species level based on shell morphological characteristics. Other relevant parameters were recorded in the human-water-contacts such as species of plants and animals associated with snails, vegetation cover, food remains, presence of excreta (feces) in the vicinity of human-water contact points.

### Cercarial releasing pattern

Collected snails were rinsed and placed individually in 24-well culture petri-dishes containing 1 ml of clear, filtered water from snail collection sites. To test whether the snails are infected, they have been exposed to indirect sunlight to induce cercarial releasing. The snails were therefore exposed for 24 h. The wells of the plates were then examined for the presence of cercariae under a dissecting microscope. Snails that did not shed cercariae on the first exposure were re-exposed on the second day. Bifurcate cercariae were used to indicate that the cercariae were of mammalian origin. The rhythm of cercarial emission from each positive snail was determined over 24 h with a count every two hours, starting from 6:00 AM. For each snail, the study was carried out over seven consecutive days to show the stability of the emission pattern. The technique used was that previously described [44]. Briefly, each infested snail was placed in a glass container with 150 ml of well water at a temperature of between 24 °C and 25 °C. Every two hours, each snail was transferred to a new container with the same volume of water. The water left in the container containing the cercariae was filtered through a Nytrel polyamide filter (25 μm mesh size). The cercariae retained on the filter were stained with a Lugol's solution, then counted under a binocular magnifying glass due to the red coloration of the cercariae.

Several cercariae released by each infested snail were stored individually on the FTA cards (QIAGEN, Hilden, Germany) and then identify by molecular targeting of the nuclear (ITS 2 and 18S rDNA) and mitochondrial (cox1) regions of DNA (19). For genetic data, genetic profiles were assigned to parasites using the haploid mitochondrial gene cox1 (first two letters) and the diploid nuclear region ITS2/18S (last four letters). These include "pure" *Sh* (*Sh* cox1_*Sh* ITS2/18S: *ShxShSh*); hybrids (*Sb/Sc* _cox1, *Sh*_ ITS2, *Sh*_18S: *Sb/ScxShSh*); (*Sh*_cox1, *Sb*_ITS2 *Sb*_18S: *ShxSbSb*); (*Sh*_cox1, *Sc*_ITS2 *Sc*_18S: *ShxScSc*).

### Data analysis

Parasitological and human-water contact data were recorded on survey forms with identifiers for each child giving the sample or individual in contact with the water. Hourly cercaria emission percentages were calculated by dividing the number of cercaria emitted per hour by the total cercaria emitted daily. Data were recorded in Microsoft Excel version 2016 (Redmond, Washington, USA). Calculations of prevalence, intensity of infection and freshwater snails’ infestation rate were performed using SPSS version 23.0 software (IBM, Chicago, Illinois, USA). Participants ages were grouped into two age categories i.e. 6–10 years old and 11–14 years old. Multivariate statistical analyses were performed to assess the relationship between sociodemographic data and HWCP parameters. For human-water contact, the comparison of percentages between sites according to sex and age was made by calculating the proportion of each sex or age group within the population of each site. The association between the presence of blood in urine and water contact activities was assessed by a logistic regression model. Differences in proportions were tested using the chi-square test or Fisher's exact test), depending on the data. *P*-values below 0.05 were considered significant.

## Results

### Parasitological data

A total of 393 urine samples were examined for *Sh* ova (Table 1). The overall prevalence was 69.2% (272/393). The prevalence and intensity of infection were significantly higher in Diakalel compared to Fangouné Bamanan (*P* < 0.0001*)*. Conversely, there was no significant difference in prevalence and intensity with respect to sex and age of the participants *(P* > 0.05).

**Table 1:** Variation in socio-demographic factors (site, sex and age) as a function of the prevalence and intensity of *Schistosoma haematobium* infection in the Kayes region, September 2021

Socio-demographic variables	Total	Prevalence			Intensity		
		Positive *n* (%)	95% *CI*	*P*-value	Low *n* (%)	High *n* (%)	*P-value*
*Sites*							
Diakalel	251	196(78.1)	72.5–83.0	< 0.0001	148(59.0)	48(19.1)	< 0.0001
Fangouné Bamanan	142	76(53.5)	45.0–62.0		61(43.0)	15(10.6)	
*Gender*							
Female	174	127 (73.0)	60.9–85.1	0.09	91 (52.3)	36 (20.7)	0.57
Male	219	145 (66.2)	51.4–80.9		118 (53.9)	27 (12.3)	
*Age, years*							
6–10	197	138 (48.4)	34.4–62.4	0.40	99 (50.3)	39(19.8)	0.13
11–14	196	134 (52.2)	38.2–66.2		110 (56.1)	24(12.2)	
Total	393	272 (69.2)	49.4–89.0		209 (53.2)	63 (16.0)	

### Characteristics of human-water contact population survey

The human-water contact activities involved 97 participants, 58 in Fangouné Bamanan and 39 in Diakalel. In terms of sex, females were more common both in Diakalel and Fangouné Bamanan (*P* = 0.042). No child aged ≤ 5 years old was observed in Diakalel. While participants aged 16 years and older were predominant in Fangouné Bamanan, those aged 6–15 years were also significantly numerous in Diakalel (*P* = 0.003) (Table 2).

**Table 2:** Gender and age distribution of the sampled participants of the human-water contact study in the two study sites in Kayes region, September 2022

Socio-demographic variables	Total	Fangouné Bamanan *n* (%)	Diakalel *n* (%)	*P-value*
Gender				
Male	18	07 (12.1)	11 (28.2)	0.042
Female	79	51 (87.9)	28 (71.8)	
Age group, years				
0–5	7	07 (12.1)	0	0.003
6–10	23	10 (17.2)	13 (33.3)	
11–15	28	12 (20.7)	16 (41.0)	
≥ 16	39	29 (50.0)	10 (25.6)	
Total	97	58 (59.8)	39 (40.2)	

### Water contact patterns and interactions

All human-water contact (HWC) activities varied significantly according to gender, age and duration of exposure (Table 3). However, the major water-contact activity in all the communities was domestic (62.9%) led by 84.6% of older females aged 16 and above. Overall, the percentage of domestic activities decreases with age, while that of recreational activities increases with age. Children under the age of 5 only engaged in recreational activities in contact with water. Among those who engaged in recreational activities, children under 10 years old were the most affected. In terms of exposure duration, most study participants, 81.4% (79/97), were in contact with snail-infested freshwater for between 6 and 30 min. During this contact, recreational activities, followed by occupational and domestic activities, were observed respectively (Table 3). Recreational activities carried out mainly by children aged 6–10 years were associated with the longest duration (60 min) of contact with infected water. The frequency of domestic activities varied based on their nature. For instance, laundry, the most common activity (82.9%), was typically done once a week, while activities such as crockery were carried out daily. Also, swimming, primarily enjoyed by children, served as a central recreational activity (Table 3).

**Table 3:** Cross-tabulation of gender, age groups, duration and frequency of activities of exposure and water-contact activities in the two study villages, September 2022

Socio-demographic variables		Human water contact activities, *n* (%)			
	Total	Recreational	Domestic	Professional	*P*-value
Gender					
Female	79	14 (17.7)	61 (77.2)	4 (5.1)	< 0.0001
Male	18	14 (77.8)	0	4 (22.2)	
Total	97	28 (28.8)	61 (62.9)	8 (8.2)	
Age group, years					
0–5	7	7 (100.0)	0	0	< 0.0001
6–10	23	12 (52.2)	9 (39.1)	2 (8.7)	
11–15	28	7 (25.0)	19 (67.9)	2 (7.1)	
≥ 16	39	2 (5.1)	33 (84.6)	4 (10.3)	
Total	97	28 (39.2)	61 (62.9)	8 (8.2)	
Duration of water-contact					
≤ 5 min	2	0	0	2 (100.0)	< 0.0001
6–14 min	26	13 (50.0)	7 (26.9)	6 (23.1)	
15–30 min	53	33 (62.3)	1 (1.9)	19 (35.8)	
≥ 60 min	16	15 (93.7)	0	1 (6.3)	
Total	97	61 (62.9)	8 (8.2)	28 (28.8)	
Water-contacts activities					
Once a week	41	34 (82.9)	7 (17.1)	0	< 0.0001
Thrice a week	1	1 (100)	0	0	
Daily	45	16 (35.6)	21 (46.6)	8 (17.8)	
Total	97	61 (62.9)	8 (8.2)	28 (28.8)	

Recreational activities: swimming, games; domestic activities (laundry, dishes, fetching water); professional activities (fishing, crossing)

**Fig. 3:**
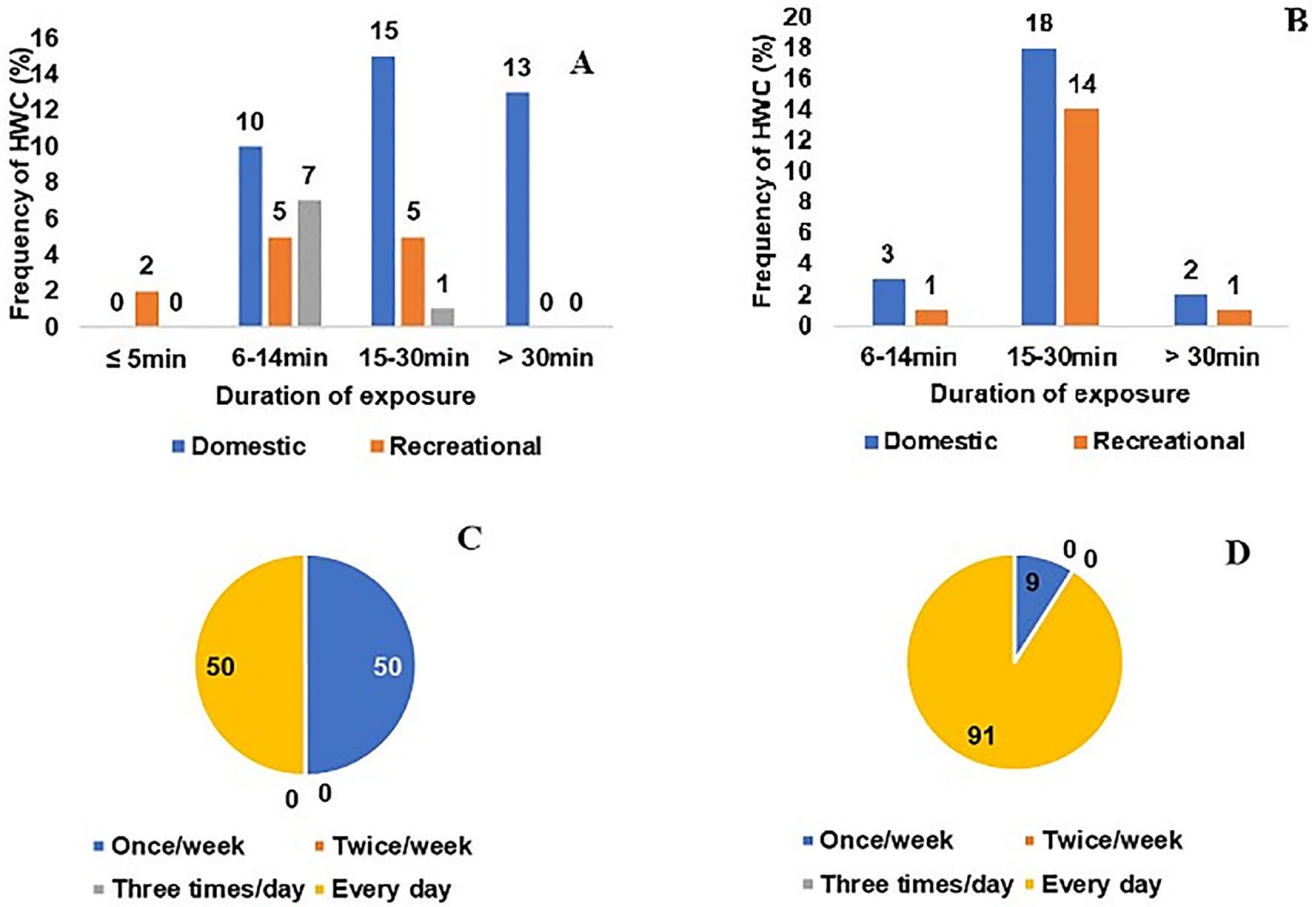
Human-water-contact activities (HWCA) frequency (%) associated to duration of exposure of the participants to surface water systems and human-water-contact (HWC) frequency associated to the exposed part of the body at both communities, September 2022. **A** Human-water-contact activities (HWCA) frequency (%) associated to duration of exposure of the participants to surface water systems at Fangouné Bamanan; **B** Human-water-contact (HWC) frequency (%) associated to the duration of exposure of participants to surface water systems at Diakalel; **C** arms/feet; **D** whole body) in children aged 6–10 years at Fangouné Bamanan and Diakalel

Figure 3A shows the variation in the duration of exposure of participants to contaminated water at Fangouné Bamanan. An overall exposure duration of 6 to 30 min accounts for up to 74.1% of all the participants. Meanwhile, domestic activities alone exposed up to 22.4% of the participants to the cercarial infested water for durations exceeding 30 min. In contrast to Fangouné Bamanan, more than 80% of the people were exposed to cercaria infested water within 15–30 min for only domestic and recreational activities (Fig. 3B). While in children aged 6–10 years, the frequency of arm/foot contact with water once a week was comparable to that of daily contact (Fig. 3C). In contrast, 90.9% (10/11) of their whole-body surface area were exposed to surface water systems every day (Fig. 3D).

### Hematuria and human-water-contact (HWC) activities

No significant association was observed between the prevalence of blood in urine and human water contact activities in this study. However, those engaged in recreational and occupational activities were 0.09 and 0.24 times more likely to develop hematuria than those engaged in domestic activities (Table 4).

**Table 4:** Cross-tabulation human-water-contact and hematuria in the two study sites, September 2022

Water-contacts activities	Total	Hematuria	*OR*	*P*-value
Domestic	60	13 (21.6)	–	
Professional	9	3 (33.3)	0.24	0.3
Recreational	28	7 (25.0)	0.09	0.054
Total	97	23		

– Not applicable

### Snail species, distribution and abundance

A total of 378 freshwater snails were collected at 14 different human/water contact point sites. Of the 378 snails, 126 were collected at HWCP-H (Human water contact point H) on the 8 along the Fangouné Bamanan stream and 252 at 3 HWCP (A, B, C) on the 6 Diakalel sites along the tributaries of the Senegal River (Table 5 and Fig. 2). All the collected snails were of the *B. truncatus* species, identified by their shell morphology. In Diakalel where two types of habitats (the river and its tributaries) were examined, snails were found only in tributaries and were collected on water lily in 7 out of 8 sites in Fangouné Bamanan. On several occasions, they were also associated with food scraps or different supports (rags, old boxes, pieces of wood, etc.) abandoned in the water. Other aquatic fauna encountered includes fry, frogs, leeches and insect larvae.

**Table 5:** Natural prevalence of shedding cercariae of *Schistosoma* spp. in Fangouné Bamanan and Diakalel, September 2022

	Fangouné Bamanan			Diakalel		
Capture site *B. truncatus*	Collected, *n*	Infected, *n*	Prevalence, %	Collected, *n*	Infected, *n*	Prevalence, *%*
A	6	1	16.7	111	7	6.3
B	1	0	–	82	1	1.2
C	7	0	–	59	0	0
D	14	0	–	–	–	–
E	9	0	–	–	–	–
F	8	0	–	–	–	–
G	74	18	24.3	–	–	–
Total	126	19	15.1	252	8	3.2

– Not applicable

**Fig. 4:**
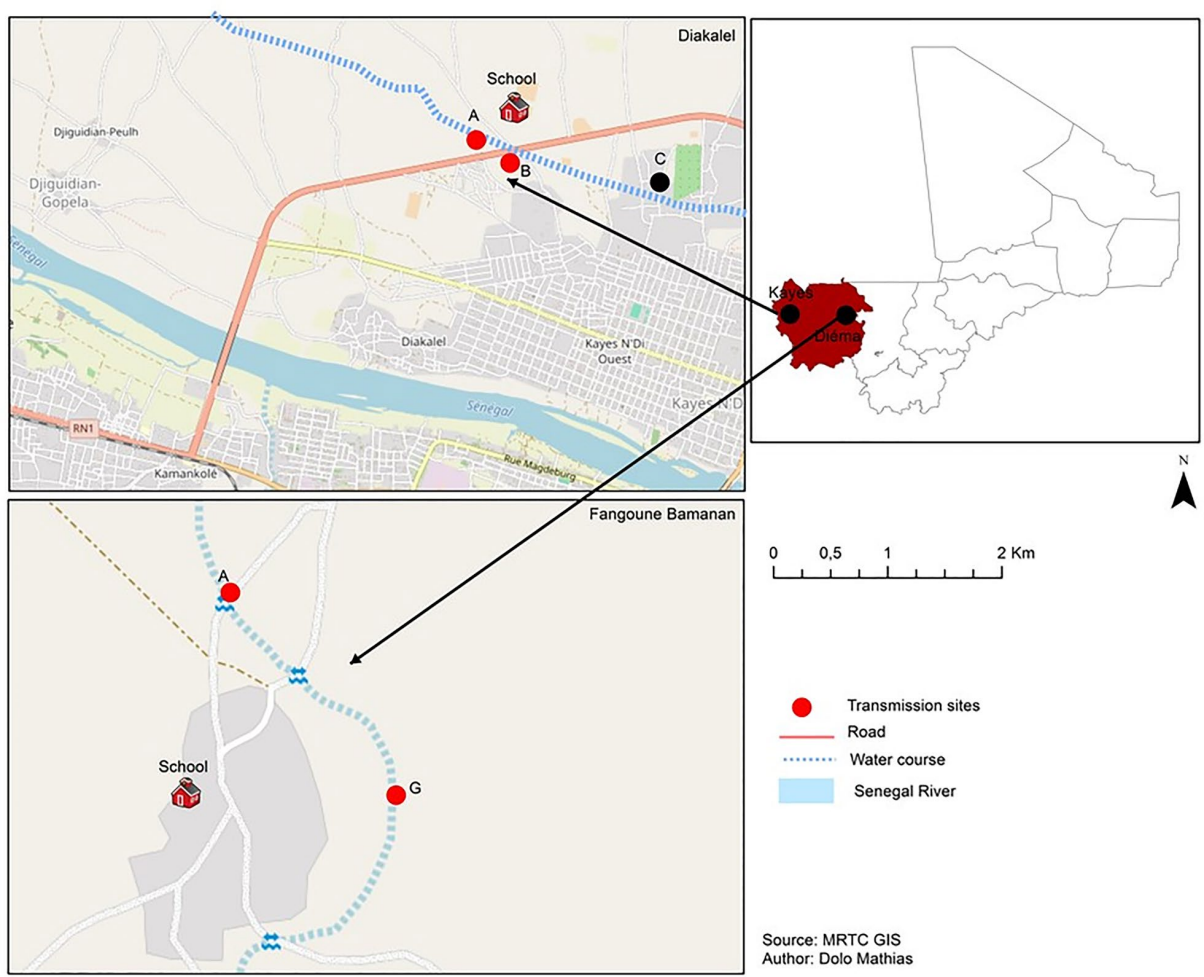
Map of human Water Contact Point (points in dark) associated to transmission sites (points in red) in the two study communities (Diakalel and Fangouné Bamanan), September 2022. The red dots (**A**, **B** in Kayes and **A**, **G** in Fangouné Bamanan) indicate the transmission sites

As expected, numerous snail samples collected during the survey harbored cercariae. Two HWCP (A and G) in Fangouné Bamanan and 2 (A and B) in Diakalel provided infected snails (Table 5, Fig. 4). Overall, 7.1% (27/378) of snails emitted *Schistosoma* cercariae. The prevalence of schistosome cercariae shedding (PSCS) was 15.0% (19/126) in Fangouné Bamanan and 3.3% (8/252) in Diakalel. Regardless of HWCP in each site, the highest PSCS was recorded at point G, 24.3% (18/74) in Fangouné Bamanan and point A, 6,1% (7/111) in Diakalel (Table 5).

### Cercarial emission patterns

**Fig. 5:**
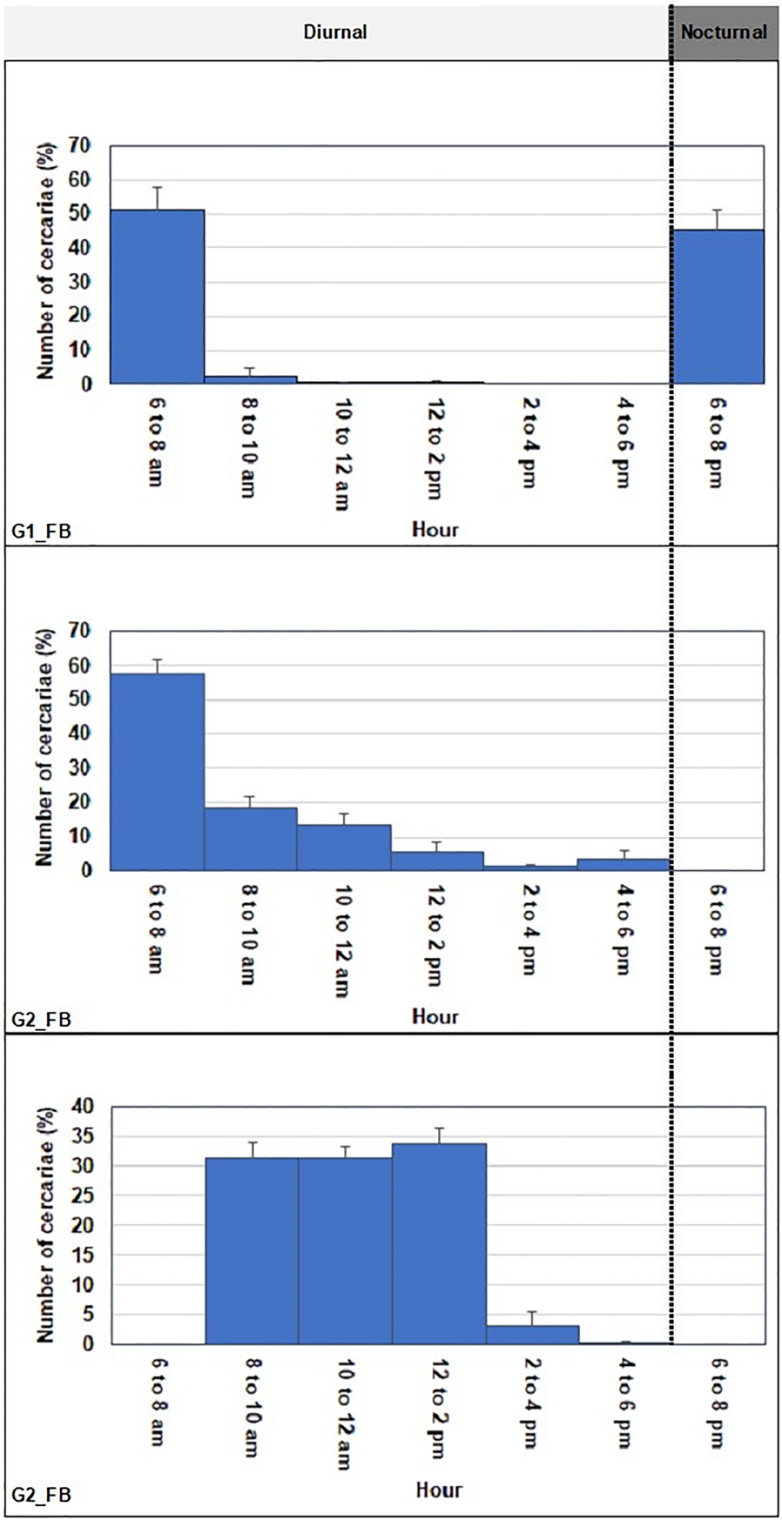
Cercarial emission patterns from *Schistosoma haematobium* naturally infected: **a** Early diurnal pattern for G2-FB; **b** Midday diurnal pattern for G3-FB; **c** Early to late nocturnal pattern for G1-FB in Fangouné Bamanan, September 2021. Gx-FB corresponds to snail x collected at point G in Fangouné Bamanan

Curves representing the average daily peak in cercarial emissions (circadian rhythm) of *B. truncatus* snails from Fangouné Bamanan are shown in Fig. 5 which shows variability in cercariae emission.We identified three distinct emission patterns at Fangouné Bamanan, with each curve representing the rhythmic cercariae emission of a group of snails sharing the same pattern:

(i) early diurnal emission was observed in 6 out of the 19 snails (31.6%). Cercaria emission commenced at 6:00 AM, coinciding with the onset of the light period, and reached its peak at 8:00 AM for G2-FB; (ii) A midday diurnal pattern was found in 11 out of the 19 snails (57.9%). The average emission peak occurred at 2:00 PM for G3-FB and (iii) a combination of early diurnal (6:00–8:00 am) and nocturnal (6:00–8:00 PM) patterns was observed in 2 snails (10.5%) out of the 19 for G1-FB.

**Fig. 6:**
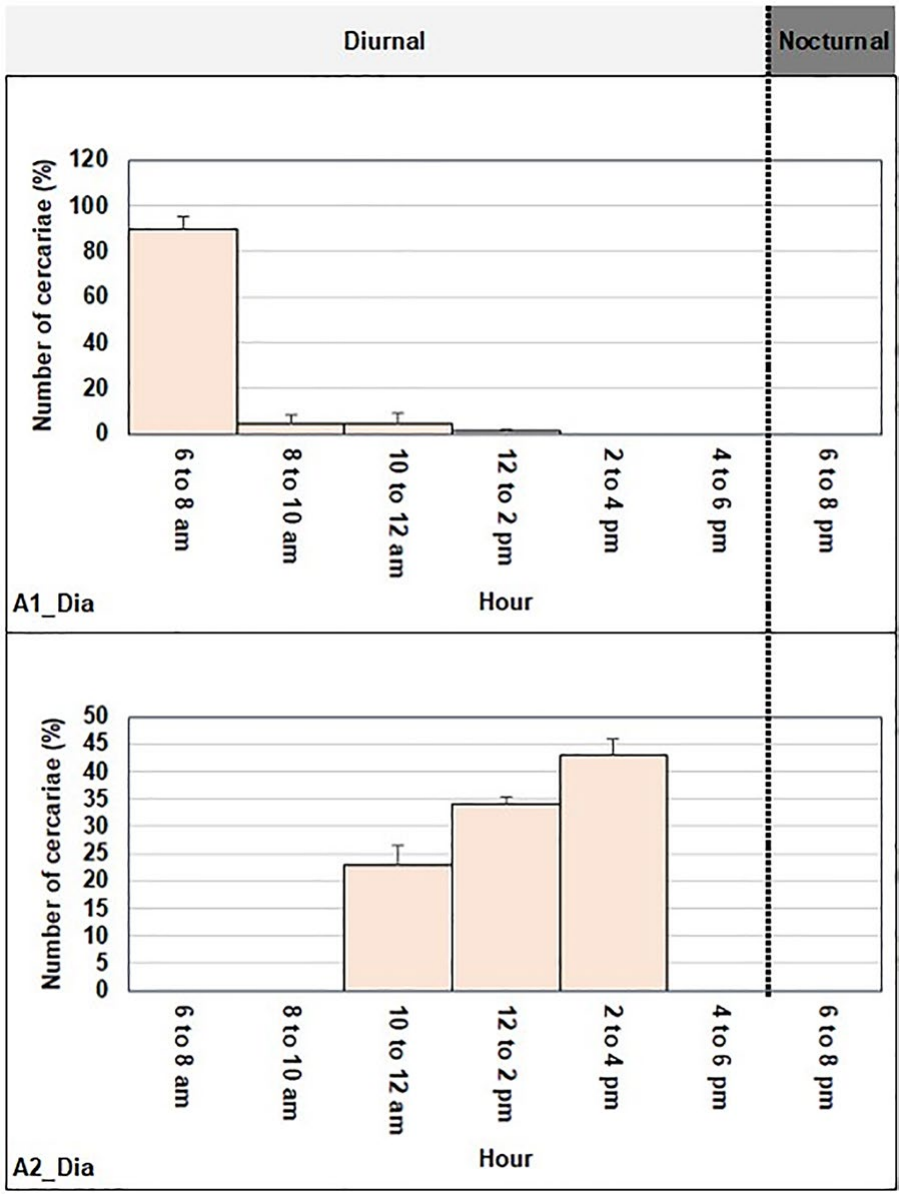
Cercarial emission from *Schistosoma haematobium* naturally infected: **a** Early diurnal pattern for A2-Dia; **b** Midday diurnal pattern for A1-Dia in Diakalel, September 2022. Ax-Dia corresponds to snail x collected at point A in Diakalel

Profiles of the average daily peak of cercarial emissions in *B. truncatus* snails from Diakalel are shown in Fig. 6. We identified two different patterns, and each snail hosted only one peak. a) An early diurnal pattern was observed for 4 of the 8 snails (50.0%). Cercaria emission peaked at 8:00 AM for A2-Dia. b) A midday diurnal pattern was found for 4 of the 8 snails (50.0%). The average emission peak at 2:00 PM for A1-Dia.

### Genotyping of schistosomes for cox1 and ITS/18S rDNA genes

The genetic profiles of cercariae released by snails in September 2021 were determined (Table 6). The profiles varied according to the period and sites of release. The cercariae released by snails collected at Diakalel gave two different profiles. Between 6:00 AM and 10:00 AM, all cercariae released were hybrids (*Sb/Sc_ShxSh*), then between 10:00 AM and 4:00 PM, cercariae were pure *Sh* (*Sh_ShxSh*) profiles. Two snails had emitted both pure and hybrid species. Similarly, at Fangouné Bamanan, the cercariae emitting patterns also showed two different profiles. Here, the pure *Sh* cercariae were found between 6:00 AM and 6:00 PM, unlike the hybrids (*Sb/Sc_ShxSh*) were emitted between 6:00 PM and 8:00 PM (Table 6). One snail had emitted both pure and hybrid species.

**Table 6:** *Schistosoma haematobium* cercariae genetic profiles described in the two study sites using RD-PCR, September 2022

Sites	Time	No. cercariae	cox1	ITS/18S rDNA	Profile
Diakalèl	6:00–8:00 AM	5	*Sb orSc*	*ShxSh*	*Sb/Sc_ShxSh*
	8:00–10:00 AM	5	*Sh*	*ShxSh*	*Sh_ShxSh*
	10:00 AM–12:00 PM	5	*Sh*	*ShxSh*	*Sh_ShxSh*
	12:00–2:00 PM	5	*Sh*	*ShxSh*	*Sh_ShxSh*
	2:00–4:00 PM	5	*Sh*	*ShxSh*	*Sh_ShxSh*
	4:00–6:00 PM	0			
	6:00–8:00 PM	0			
Total		25			
Fangouné Bamanan	6:00–8:00 AM	5	*Sh*	*Sh*	*Sh_ShxSh*
	8:00–10:00 AM	5	*Sh*	*Sh*	*Sh_ShxSh*
	10:00 AM –12:00 PM	5	*Sh*	*Sh*	*Sh_ShxSh*
	12:00–2:00 PM	5	*Sh*	*Sh*	*Sh_ShxSh*
	2:00–4:00 PM	5	*Sh*	*Sh*	*Sh_ShxSh*
	4:00–6:00 PM	5	*Sh*	*Sh*	*Sh_ShxSh*
	6:00–8:00 PM	5	*Sbor Sc*	*Sh*	*Sb/Sc_ShxSh*
Total		35			

## Discussion

This study was conducted in the Senegal River Basin in Mali to examine the interactions between three essential elements of schistosomiasis transmission cycle i.e., the definitive host (human or animals), the freshwater snail (intermediate host) and the surface water systems where the hosts meet during various water contacts activities. Epidemiological data on schistosomiasis prevalence in human and snail intermediate hosts is crucial for identifying transmission sites as well as the parasite’s distribution in a particular area and informing decision-makers and control programs. With reference to the current integrative methods of control and elimination of schistosomiasis, the current WHO roadmap sets goals to eliminate schistosomiasis as a public health problem by 2030 [45]. Therefore, an in depth understanding of the disease context in sub-Saharan Africa with respect to ongoing transmission in endemic zones is a requirement to achieve these set goals. In this study, 69.2% of the schoolchildren were infected with *Sh*. The high prevalence observed is consistent with findings reported in the same area where the prevalence of urinary schistosomiasis was 84.4% in preschool-aged children especially in Fangouné Bamanan [37]. Our findings were consistent with a previously reported prevalence of 72.4% observed at the Office du Niger, Central Mali [7] but higher than 14.0% that was recorded in Côte d’Ivoire [5].

The interactions between humans and freshwater sources play a significant role in influencing schistosomiasis transmission, especially when considering the specific type of water-contact activities. The direct dependence on sources of unhealthy water, i.e. surface water, increases the probabilities of exposure to contaminated water thus, the risk of *Sh* infection. In this study, we found that people of Fangouné Bamanan and Diakalel are highly dependent at a given time on surface water from the streams and Senegal river, respectively for their domestic, professional, or recreational needs. Consequently, these freshwater sources widespread utilization for various purposes including washing, laundry, fetching for domestic cooking, fishing, river crossing, animal watering, bathing, swimming, and recreational activities. Although sanitation facilities generally exist in both communities, their low use was the key factor driving community members, especially children, to engage in open defecation (data do not present here). In this study, a common practice observed was the unsanitary disposal of urine and feces, which resulted in the contamination of water bodies with schistosome eggs. Proximity to these freshwater bodies has also play a significant role in influencing unsanitary practices by the inhabitants of the study communities: for instance, several houses Fangouné Bamanan community are built at the bank of the stream with toilet facilities built at the riverbank. This setup provides a more comfortable and well-ventilated environment for open defecation, which, unfortunately discourages the use of latrines. This is therefore consistent with Schmidlin (2013) [46] where it was reported that poor hygiene and sanitation linked to the practice of insanitary disposal of urine or feces play crucial roles in the transmission cycle of schistosomes, as eggs are released into waterbodies via excreta). Larvae resulting from eggs hatching infect the snails (intermediate hosts), which in turn release the parasites to infect humans [47, 48]. Water contamination by human urine is the initial step in the infection of *Bulinus* snails by miracidia. Subsequently, this contaminated water serves as the source of infection for individuals who come in contact it, while the risk of contamination is also increased by the duration ranging between six and thirty minutes of exposure and account for 74% of participants. In retrospect, the penetration time for cercariae was estimated to be less than 10 min for *Sm* [49], it is noteworthy that the exposure time for 71% of participants exceeded 15 min with some exceeding 60 min, this extended exposure duration is considered sufficient for cercarial penetration.

The type of water-contact activity also plays an invaluable role in the transmission of the schistosome. In this study, domestic activities were predominantly practiced by female (87%), followed by recreational activities practiced mainly by children aged 6–14 years old (67%). In fact, it has been recognized that the frequency and duration of the water-contact are influenced by the type of water-contact activity, which in turn correlates with the level exposure. Whilst, domestic water-contact activities are linked to more frequent (but rather short) water-contact activities, recreational water-contact activities occur less frequently but usually for longer durations. In our study recreational activities such as swimming facilitates exposure for longer durations, the same activity also was positively correlated with longer water-contact durations in the Shinyanga District of Tanzania, thereby increasing the exposure to schistosomiasis [50]. Similar patterns were also observed in Senegal where exposure of women and children to cercariae was influenced by the frequency and duration of water-contacts [51]. This indicates that the type of water interaction is an important factor mediating exposure to cercariae and the risk of schistosomiasis. As swimming activities usually involve longer water-contact durations as well as full submersion of the body, it is not surprising that recreational and domestic activities are significantly associated with the presence of blood in urine. However, in contrary to the what was reported in Lower Densu River basin in Ghana, more frequent water-contacts (more than twice per week) and longer water-contact durations (more than 30 min) did not show significant increase in the odds of hematuria [6]. Similarly, a study in Nigeria also highlighted that direct water contact exposes the individuals to the cercariae and thus places them at risk of infection especially those who directly depended on freshwater as a source of livelihood [52]. Similarly, fetching water for household use and other similar activities that involve frequent water-contacts, were determinant to expose children with relatively high levels of exposure to cercariae in the Densu Basin in Ghana [53]. Domestic water-contacts, such as washing clothes or kitchen utensils, and recreational water-contacts such as swimming were recorded as the main exposure factors in our two study sites. These results are supported by recent studies that showed WASH or sex is less influential risk factor for infection than water contact regarding the magnitude of the association between exposure and schistosome infection [53]. These studies revealed that having any water contact was associated with 3.14 times higher odds of infection compared to no water contact. It is therefore evident that water contact is common and, in many cases, unavoidable.

Examination of 126 snails showed that *B. truncatus* was the only snail intermediate host of human schistosomiasis encountered. In contrast to these results, the malacological fauna is richer in the rice irrigated area of Office du Niger or at the suburban area of Bamako, including other vector species such as *B. globosus* for *Sh* and *Biomphalaria pfeifferi* for *Sm* [23–25]. In our study, all the host snails were found only in the streams in Fangouné Bamanan and in the Senegal River tributaries in Diakalel because of the slow flow in these water surface abundantly covered with aquatic plants (*Pistia stratiotes* and *Nymphaea micrantha*), grasses or bushes in the riverbed. In the Senegal river however, the intense water current, waves and lack of vegetation cover prevent any snail settlement. The global natural prevalence of shedding schistosome cercariae of *Schistosoma* spp. was 7.1% (27/378) synonymous with the existence of intense outbreaks of parasite transmission. The prevalence of *Schistosoma* spp. infection in the snails was higher in Fangouné Bamanan (15.0%) than in Diakalel (3.1%) and those recorded in Bamako (8.3%) [18] but lower than those observed in Office du Niger (up to 24%) [25]. In the Niger River Valley (NRV) in Niger, the prevalence of *Schistosoma* spp. infection was low for *B. forskalii* with 0.2% (24/11,989), also low in *B. truncatus* (0.8%, 342/42,500) and relatively high in *Biomphalaria pfeifferi* (3.4%, 79/2290) [54]. Despite the large number of snails collected and the high number of sites surveyed in the Niger valley, infection rates remain low compared with our previous results. These results observed in the Niger Valley and elsewhere [55] show that natural infestation rates are generally low. Careful selection of the water contact point where snails are caught, i.e. where they are most likely to be infested by excreta (urine and feces), has more influence on the snail infestation rate than a high number of samples caught, or sites surveyed. In other words, isolated HWCP that are seldom frequented by the population can provide many samples, almost all of which will be negative. In our study, beyond of the intensity of water contact activities, the village of Fangouné, for example, is located almost on the riverbed, offering young children the opportunity to defecate there, given the difficulties of accessing traditional toilets that are less comfortable for them.

Our results on the chronobiology of *Schistosoma* spp. cercarial emission in Fangouné Bamanan and Diakalel in the Kayes region showed that the rhythm of emergence was of a circadian type.

The first pattern, early diurnal peaking between 6:00 AM and 8:00 AM, exhibited an hybrid cercarial emission pattern; it was observed in 6 out of 19 *B. truncatu*s examined (31.6%) (for G2-FB in Fangouné Bamanan; A2-Dia in Diakalel).To buttress this, such a pattern was also observed in *Sb* from Benin [56], Sardinia (Italy), Sudan and Spain [57] and Niger [58]. The difference between the genetic profiles could be explained by the nature of the molecular tools used to identify the nuclear gene, i.e. ITS in previous studies and ARMS in our study. The second pattern was observed for 47.4% of snails from 9 *B. truncatus* (for G3-FB in Fangouné Bamanan; A1-Dia in Diakalel; Fig. 5 & 6) with cercariae emitting between 10:00 AM and 6:00 PM released during daylight hours. It was similar to what has been published on *Sh* in humans from Algeria [59], Morocco [29], Niger [57], and Gabon [31]. The third pattern corresponding to a typical early pattern for *Sh* accompanied by a diurnal pattern for *Sb/Sc_ShxSh* emergence peaking around 3:00 PM and 7:00 PM respectively, was found for 2 of 19 snails (10.5%) (for G1-FB in Fangouné Bamanan). Such double peak in cercarial emergence was reported for *Sb* for the first time in Benin [23]. For another animal schistosome, *S. margrebowiei*, two emergence peaks per day were described, with the first peak occurring 1 h after the onset of daylight, and the second peak one hour after the onset of darkness [60]. On the other hand, many authors support that the cercarial emergence behavior is of genetic origin, [61]. Thus, even if the schistosome is subjected to different environmental pressures at the level of its intermediate snail host, its behavior remains unchanged. It is the case of the emission profile of *Sh* from the snail *B. truncatus* which does not change when the snail is also infected with another species of schistosome, *Sb* [29]. Regardless to the behavior genetic supporting, the two-peak cercarial emergence observed in our study could be assigned to the same species *Sb* as demonstrated in Benin [56], or to two different species of which the second one remains to be identified at the molecular level. In the latter case, the two-peak cercarial emergence found in a single snail sample suggests that, clearly, two miracidia succeeded in developing in this one snail, leading to the double peak in cercarial emergence. This is the case of our study where the only snail has been infected with miracidia of *Sb*, *Sc* and *Sh* leading to the hybrid *Sb/Sc_ShxSh*.

From an evolutionary point of view, emergence times are usually well correlated with times when the definitive putative hosts species are present in the water and available for infection [62]. In Diakalel and Fangouné Bamanan, the circadian rhythm of emissions, with a peak around 3:00 PM, can be explained by human contact with water, which is essentially related to bathing during the hottest hours of the day and for domestic activities such as laundry and washing kitchen utensils at any time of the day. In the particular case of Fangouné Bamanan a rural area, the practice of activities other than bathing, such as artisan fishing, can result in a change in the cercarial emission pattern to a very particular pattern, with a primary peak occurring around 3:00 PM but secondary peaks at dawn and dusk, when fishers (school aged children and young adults) are in contact with water, as has been shown in *Sm* in Benin [63].

Regarding the limitations of our study, the results obtained were generated following surveys carried out as part of one round cross-sectional study. Considering potential spatial and temporal variations in malacological and human-water contact, data surveys must be multiplied over at least two or three years. For a study which relies on self-reported cases of blood in urine, some degree of reporting bias must be expected, particularly, the effects of sex and age must be treated with great prudence. Although all children engage in multiple water-contact activities, however, only the predominant ones were reported. This may affect the respective effects of the individual water-contact activities. The study design assumes that all children have some degree of exposure, therefore there was no control group, thus not allowing robust case-control analysis. Hybrid strains were identified, but a relatively high number of at least a hundred cercaria could give better results about the hybrid cercariae observed. We assume that a technical roadmap supporting the coherence of the document could be drawn by organically combine the three parts (human water contact findings, malacological data and Cercarial chronobiology).

## Conclusions

This study evaluated human/water contact and its influence on the patterns and genetic profile of cercariae emitted by snails, intermediate hosts of urogenital schistosomiasis. Our results suggest that in Mali, domestic activity, which seems to be carried out only by women, was the main factor predisposing to schistosomiasis infection, followed by recreational activity practised mainly by children. The infection risk in the populations was the presence of infected snails (*Bulinus truncatus* and *B. globosus*) combined with a chronobiological polymorphism in the cercarial emergence rhythm released from these snails, a consequence of the contamination of the water by human excrement (urine or stool). The cercarial emissions of snails naturally infected are observed at the early and middle of the day (in Diakalèl and Fangouné Bamanan) and also in the first two hours of the night in Fangouné Bamanan. The molecular data from cercariae collected at Fangouné Bamanan showed a unique *S. haematobium* profile with a chronobiological polymorphism suggesting (i) an adaptation of the parasite to the time of human (or animal) host water contact or (ii) an opening up of the host infection spectrum by the parasite in order to increase their survival, which is a consequence of hybridization between human and animal schistosome species. Further studies on animal reservoir hosts such as domestic livestock and small commensal mammals such as rodents in these sites could provide more complete information on the dynamics of water contact activities that could help to better explain certain chronobiological profiles that we have observed. These data could help to adapt local measures for sustainable control of the disease.

## Data Availability

Not applicable.

## Abbreviations

HWC: Human-water contact
RD: Rapid diagnostic
ARMS: Amplification refractory mutation system
PCR: Polymerase chain reaction
ITS: Internal transcribed spacer
*Sh*: *Schistosoma haematobium*
*Sb*: *Schistosoma mansoni*
*Sc*: *Schistosoma curassoni*
MDA: Mass drug administration
WHO: World health organization
HWCP: Human water-contact points
GPS: Global positioning system
PSCS: Prevalence of schistosome cercariae shedding
